# Westernization of lifestyle affects quantitative and qualitative changes in adiponectin

**DOI:** 10.1186/s12933-017-0565-z

**Published:** 2017-07-06

**Authors:** Mitsunobu Kubota, Masayasu Yoneda, Norikazu Maeda, Haruya Ohno, Kenji Oki, Tohru Funahashi, Iichiro Shimomura, Noboru Hattori

**Affiliations:** 1 Department of Molecular and Internal Medicine, Graduate School of Biomedical & Health Sciences, Hiroshima University, 1-2-3 Kasumi, Minami-ku, Hiroshima, 734-8551 Japan; 2 Department of Metabolic Medicine, Graduate School of Medicine, Osaka University, Osaka, Japan; 3 Department of Metabolism and Atherosclerosis, Graduate School of Medicine, Osaka University, Osaka, Japan

**Keywords:** Lifestyle westernization, Japanese migration, Insulin resistance, Total adiponectin, C1q-adiponectin

## Abstract

**Background:**

Although Japanese–Americans and native Japanese share the same genetic predispositions, they live different lifestyles, resulting in insulin resistance in Japanese–Americans. We investigated whether the quantitative and qualitative changes in adiponectin (APN) due to differences in lifestyle contribute to the development of insulin resistance.

**Methods:**

We evaluated 325 native Japanese in Hiroshima, Japan and 304 Japanese–Americans in Los Angeles, the United States, who were aged between 30 and 70 years and underwent medical examinations between 2009 and 2010. All participants underwent a 75-g oral glucose tolerance test (OGTT) to assess their glucose tolerance. The insulin response to oral glucose load, the Matsuda index, total APN levels, and C1q-APN/total-APN ratios were compared between native Japanese and Japanese–Americans.

**Results:**

Compared with the native Japanese, the Japanese–Americans had significantly lower Matsuda index and higher area under the curve values for serum insulin concentration during OGTT in the normal glucose tolerance (NGT) and impaired glucose tolerance (IGT) groups, but not in the diabetes mellitus (DM) group. Furthermore, the Japanese–Americans had significantly lower total APN levels and higher C1q-APN/total-APN ratios than the native Japanese in the NGT and IGT groups, but not in the DM group.

**Conclusions:**

This study suggested that, in Japanese people, the westernization of their lifestyle might affect quantitative and qualitative changes in APN and induce insulin resistance.

**Electronic supplementary material:**

The online version of this article (doi:10.1186/s12933-017-0565-z) contains supplementary material, which is available to authorized users.

## Background

Adiponectin (APN) is an adipose-specific protein which is exclusively secreted from adipose tissue into the peripheral blood. Circulating APN is considered to be a cardiometabolic marker associated with global cardiovascular risk [1]. Hypoadiponectinemia is closely associated with the risk for insulin resistance [2, 3], metabolic syndrome [4] and coronary artery disease [5, 6].

APN and complement C1q have homologous structures [7, 8]. A previous study showed that APN inhibits C1q-induced arthritis in murine arthritis models [9]. Although APN is known to bind with C1q and several other molecules in vitro [10, 11], the in vivo mechanism of APN action has not been sufficiently elucidated. Nakatsuji et al. discovered that APN forms a protein complex with C1q in human serum [12]. Furthermore, the ratio of C1q-APN complex to total APN (C1q-APN/total-APN ratio) may be a more sensitive marker of metabolic syndrome and arteriosclerotic diseases than the total APN level alone [12–14].

Since 1970, we have conducted a medical survey, the Hawaii–Los Angeles–Hiroshima Study, targeting Japanese immigrants from Japan to the United States and their descendants [15]. Because Japanese–Americans, while genetically equivalent to native Japanese, have a different lifestyle, they are an appropriate population for investigating the effects of environmental changes on lifestyle-related metabolic diseases among Japanese people. In comparison with native Japanese living a Japanese lifestyle in Hiroshima, insulin resistance is more severe [16], and the prevalence of diabetes mellitus (DM) and metabolic syndrome is higher [17, 18] in Japanese–Americans living an American lifestyle in Hawaii or Los Angeles. We also reported that a low level of serum APN was a significant risk factor for type 2 DM [19]. Because decreased APN is associated with insulin resistance [2, 3], APN may be involved in insulin resistance induced by the westernization of lifestyles in Japanese people.

We hypothesized that not only the serum APN concentration but also the C1q-APN/total-APN ratio contributes to the higher degree of insulin resistance in Japanese–Americans compared with native Japanese. In this study, we compared insulin response to an oral glucose load, total APN levels, and C1q-APN/total-APN ratios between native Japanese and Japanese–Americans in order to determine the impact of the environmental factor of lifestyle on the quantitative and qualitative changes in APN.

## Methods

### Study subjects

This study included 325 native Japanese living in Hiroshima, Japan and 304 Japanese–Americans living in Los Angeles, USA, who were aged between 30 and 70 years and underwent medical examinations between 2009 and 2010. Those under drug treatment for DM or dyslipidemia, those with renal failure (an estimated glomerular filtration rate of <30 mL/min/1.73 m^2^ according to the Japanese equation [20]), and those with a C-reactive protein (CRP) level ≥10 mg/L were excluded. All participants underwent a 75-g oral glucose tolerance test (OGTT). This study was conducted with the approval of the Ethics Committee of Hiroshima University.

### Anthropometric data and laboratory tests

After overnight fasting, each participant was interviewed, provided written informed consent, and underwent a physical examination and venous blood collection. Body measurements were taken in the standing position. Body mass index (BMI) was calculated as weight (kg)/height (m^2^). Each blood sample was centrifuged, immediately frozen, and stored until measurement. Serum glucose levels were measured using the hexokinase method. Immunoreactive insulin (IRI) levels were measured using the double-antibody radioimmunoassay. Total cholesterol and triglyceride levels were measured using enzymatic assays. High-density lipoprotein (HDL) cholesterol levels were measured using a homogeneous assay. Low-density lipoprotein (LDL) cholesterol was calculated using the Friedewald equation [21]. CRP levels were measured using the highly sensitive latex agglutination method. Total APN levels were measured using an enzyme-linked immunosorbent assay (ELISA) (human adiponectin ELISA kit; Otsuka Pharmaceutical Co., Tokushima, Japan). C1q-APN levels were measured using a previously reported ELISA method developed in a joint study by Osaka University and Otsuka Pharmaceutical Co. [12]. DM was diagnosed as a fasting serum glucose level ≥126 mg/dL (7.0 mmol/L) or a serum glucose level ≥200 mg/dL (11.1 mmol/L) at 2 h after OGTT. Normal glucose tolerance (NGT) was defined as a fasting serum glucose level <110 mg/dL (6.1 mmol/L) and a serum glucose level <140 mg/dL (7.8 mmol/L) at 2 h after OGTT. Impaired glucose tolerance (IGT) was diagnosed in participants who did not meet the criteria of either NGT or DM [22]. Smoking status was assessed by self-report.

The Matsuda index and the area under the curve (AUC) for serum IRI during OGTT (OGTT_AUC_ IRI) were used as insulin resistance indices. The Matsuda index was calculated using serum glucose and insulin levels at 0, 60, and 120 min after OGTT [23, 24]. The OGTT_AUC_ IRI was determined according to the trapezoidal method from insulin levels at 0, 60, and 120 min after OGTT.

### Statistical analyses

Because of the skewed distribution of data on triglycerides, CRP, Matsuda index, OGTT_AUC_ IRI, total APN, and C1q-APN/total-APN ratios, these parameters were logarithmically transformed and analyzed. Continuous variables were compared by Student’s *t* test between two categories, or analysis of variance (ANOVA) among three categories; if they were found to be significant, the Tukey–Kramer method was used to assess the association between categories. Categorical variables were analyzed using *χ* ^*2*^ tests. Pearson’s correlation analysis and multiple regression analysis were performed to determine the associations between insulin resistance and the total APN level or the C1q-APN/total-APN ratio. *P* values <0.05 were considered statistically significant. All analyses were performed using IBM SPSS Statistics for Windows, version 20.0 (IBM Corp., Armonk, NY, USA).

## Results

The baseline characteristics of the participants are shown in Table 1. The male-to-female ratio, age, and BMI were comparable between native Japanese and Japanese–Americans. The systolic blood pressure, diastolic blood pressure, and triglyceride and CRP levels were significantly higher, whereas the Matsuda index was significantly lower in the Japanese–Americans than in the native Japanese. Furthermore, the total APN levels were significantly lower, whereas the C1q-APN/total-APN ratios were significantly higher in the Japanese–Americans than those in the native Japanese.

**Table 1:** Baseline characteristics of participants

	Native Japanese	Japanese–Americans
N (male/female)	325 (124/201)	304 (134/170)
Age (years)	55.3 ± 11.5	55.5 ± 10.8
BMI (kg/m²)	23.0 ± 2.99	23.2 ± 3.22
Smoking (none/ex/current)	226/53/46	172/81/51*
SBP (mmHg)	124.4 ± 17.7	129.2 ± 18.1*
DBP (mmHg)	77.6 ± 11.1	82.5 ± 11.7*
Total cholesterol (mg/dL)	208.8 ± 34.4	212.8 ± 36.1
HDL cholesterol (mg/dL)	62.0 ± 14.5	61.1 ± 16.2
LDL cholesterol (mg/dL)	127.7 ± 31.5	129.7 ± 33.7
Triglyceride (mg/dL)^a^	88.0 (62.0‒124.0)	109.5 (77.3‒156.8)*
CRP (mg/L)^a^	0.31 (0.17‒0.65)	0.46 (0.24‒1.06)*
Glucose category (NGT/IGT/DM)	247/67/11	237/39/28*
Fasting glucose (mg/dL)	94.1 ± 11.4	91.8 ± 20.1
2-h glucose (mg/dL)	119.6 ± 35.4	118.8 ± 57.3
Matsuda index (mU/L)^a^	7.35 (4.84‒9.98)	6.06 (3.93‒8.66)*
Total APN (μg/mL)^a^	8.84 (5.84‒11.6)	8.15 (5.50‒11.3)*
C1q-APN/total-APN ratio^a^	8.96 (7.12‒11.6)	10.2 (7.73‒13.2)*

Data are presented as number, mean ± SD or median (25th‒75th percentile levels)

*BMI* body mass index, *SBP* systolic blood pressure, *DBP* diastolic blood pressure, *HDL* high-density lipoprotein, *LDL* low-density lipoprotein, *CRP* C-reactive protein, *NGT* normal glucose tolerance, *IGT* impaired glucose tolerance, *DM* diabetes mellitus, *APN* adiponectin

* *P* < 0.05 native Japanese vs. Japanese–Americans

^a^Parameters were transformed logarithmically before analysis

**Fig. 1:**
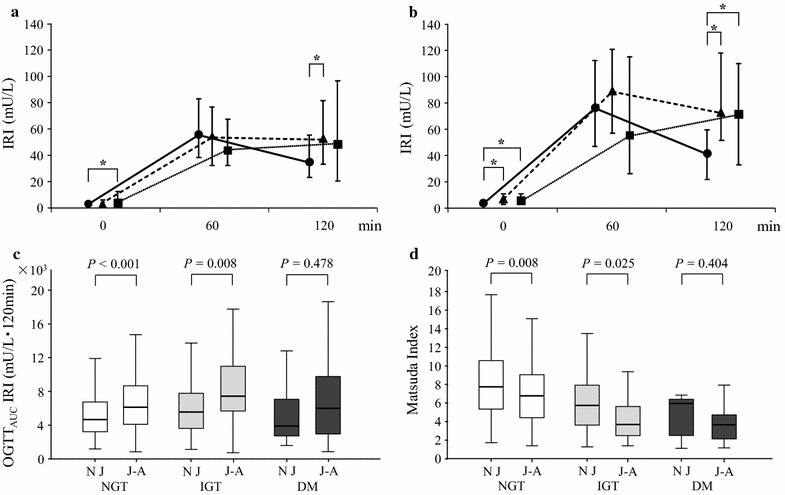
Insulin response to oral glucose load and insulin resistance in native Japanese and Japanese–Americans. Serum IRI levels during OGTT in native Japanese (**a**) and Japanese–Americans (**b**). The NGT group is indicated by *dots* and *solid lines*, the IGT group as *triangles* and *bold dotted line*, and the DM group as *squares* and *thin dotted line*. *P < 0.05 compared with NGT group. Summary of OGTT_AUC_ IRI values (**c**) and Matsuda index (**d**) by glucose tolerance status between native Japanese and Japanese–Americans. The *line* in the *middle of the box* indicates the median value; the box extends from the 25th–75th percentiles. *IRI* immunoreactive insulin, *OGTT*_*AUC*_ *IRI* area under the curve for serum IRI during OGTT, *NJ* native Japanese, *J–A* Japanese–Americans, *NGT* normal glucose tolerance, *IGT* impaired glucose tolerance, *DM* diabetes mellitus

The serum insulin concentrations at three points during OGTT according to glucose tolerance status are shown in Fig. 1. In the native Japanese (Fig. 1a), the fasting IRI (FIRI) value in the DM group and the 120-min IRI value after OGTT (120 min IRI) in the IGT group were significantly higher than those in the NGT group (FIRI in DM, *P* = 0.004; 120 min IRI in IGT, *P* < 0.001). In the Japanese–Americans (Fig. 1b), the FIRI values in the IGT and DM groups and 120 min IRI values in the IGT and DM groups were significantly higher than those in the NGT group (FIRI in IGT, *P* = 0.003; FIRI in DM, *P* = 0.008; 120 min IRI in IGT, *P* < 0.001; 120 min IRI in DM, *P* < 0.001). In order to quantify the insulin response to oral glucose load, OGTT_AUC_ IRI values were compared between the native Japanese and the Japanese–Americans according to glucose tolerance status (Fig. 1c). In the NGT and IGT groups, but not in the DM group, OGTT_AUC_ IRI values were significantly higher in the Japanese–Americans than in the native Japanese. Furthermore, in the NGT and IGT groups, but not in the DM group, the Matsuda index was significantly lower in the Japanese–Americans than in the native Japanese (Fig. 1d).

**Fig. 2:**
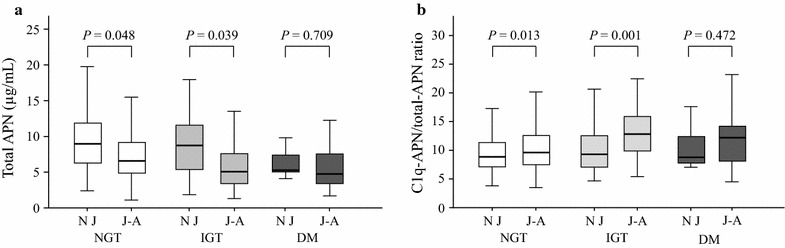
Summary of total APN level and C1q-APN/total-APN ratio by glucose tolerance status between native Japanese and Japanese–Americans. The *line in the middle of the box* indicates the median value; the box extends from the 25th–75th percentiles. *APN* adiponectin, *NJ* native Japanese, *J–A* Japanese–Americans, *NGT* normal glucose tolerance, *IGT* impaired glucose tolerance, *DM* diabetes mellitus

Next, the total APN levels and the C1q-APN/total-APN ratios were compared between native Japanese and Japanese–Americans according to glucose tolerance status (Fig. 2). In the NGT and IGT groups, but not in the DM group, the total APN levels were significantly lower in the Japanese–Americans than in the native Japanese (Fig. 2a), whereas the C1q-APN/total-APN ratios were significantly higher in the Japanese–Americans than in the native Japanese (Fig. 2b).

**Fig. 3:**
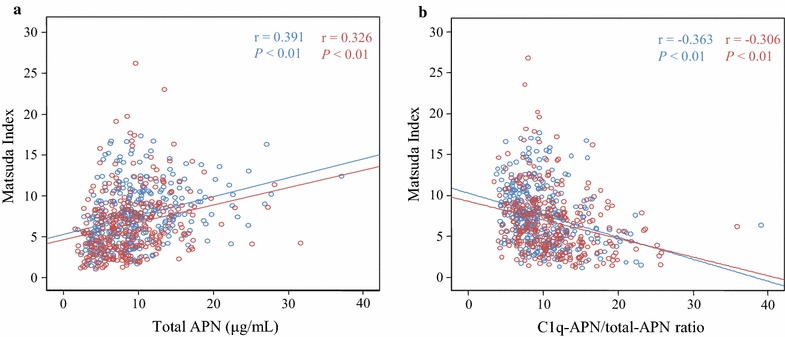
Correlations between Matsuda Index and total APN level (**a**) or C1q-APN/total-APN ratio (**b**) in native Japanese (*blue*) and Japanese–Americans (*red*). The correlations between Matsuda Index and total APN level or C1q-APN/total-APN ratio were analyzed by Pearson’s correlation analysis after logarithmic transformation

Finally, the associations between insulin resistance and the total APN level or the C1q-APN/total-APN ratio were investigated in each Japanese cohort. The Matsuda index was significantly correlated with the total APN level in the native Japanese (r = 0.391, *P* < 0.01) and in the Japanese–Americans (r = 0.326, *P* < 0.01) (Fig. 3a), and it was significantly correlated with the C1q-APN/total-APN ratio in the native Japanese (r = −0.363, *P* < 0.01) and in the Japanese–Americans (r = −0.306, *P* < 0.01) (Fig. 3b). Furthermore, multiple regression analyses revealed that the total APN level was a significantly positive explanatory factor for the Matsuda index after adjusting for age, sex, smoking status, BMI, and glucose tolerance status in the native Japanese (β = 0.305, *P* < 0.001) and in the Japanese–Americans (β = 0.218, *P* < 0.001), whereas the C1q-APN/total-APN ratio was a significantly negative explanatory factor for the Matsuda index after adjusting for the same variables in the native Japanese (β = −0.262, *P* < 0.001) and in the Japanese–Americans (β = −0.164, *P* = 0.002) (Additional file 1).

## Discussion

In the 2009–2010 medical survey, insulin resistance, total APN level, and C1q-APN/total-APN ratio, which is a new potential biomarker based on C1q-APN levels, were compared between two cohorts of Japanese people who shared the same genetic predispositions but lived different lifestyles; that is, Japanese–Americans living an American lifestyle in Los Angeles and native Japanese living a Japanese lifestyle in Hiroshima. The results of this study indicate that the westernization of lifestyles in Japanese may be associated with insulin resistance through the influence of APN abnormalities.

### Insulin resistance and APN abnormalities

At first, we investigated the patterns of insulin response to oral glucose load according to glucose tolerance status in both Japanese cohorts. Ethnic differences in insulin secretion and insulin sensitivity have been reported between Japanese and Caucasians [25]. In the present study, we revealed that the total serum insulin concentration during OGTT was significantly higher in the Japanese–Americans than in the native Japanese in the NGT and IGT groups but not in the DM group.

Subsequently, we measured serum concentrations of total APN and C1q-APN to investigate not only the quantity (total APN level) but also the quality (C1q-APN/total-APN ratio) of APN in both Japanese cohorts. As a result, both total APN levels and C1q-APN/total-APN ratios were significantly associated with the Matsuda index in the native Japanese and also in the Japanese–Americans. This indicates that the quantitative and qualitative changes in APN could relate insulin resistance in Japanese people regardless of their living countries.

However, compared with the native Japanese, the Japanese–Americans had lower total APN levels and higher C1q-APN/total-APN ratios. Furthermore, the analyses according to glucose tolerance status showed significant differences between these cohorts except for those in the DM group. The impact of the differences in lifestyles on insulin resistance and APN abnormalities appeared to have been clearly exhibited in the NGT and IGT groups. The possible reasons for the lack of a significant difference in the DM group include the potential difficulty in detecting changes in APN levels owing to lifestyle alone because APN are strongly affected by DM in both native Japanese and Japanese–Americans and the possibility that the lifestyle of native Japanese with DM is becoming similar to that of Japanese–Americans.

### Inflammation and APN abnormalities

Next, the association between chronic low-grade inflammation and insulin resistance has been reported [26]. In fact, serum CRP levels were higher in the Japanese–Americans than in the native Japanese in the present study (Table 1). In addition, total APN levels are reportedly lower in severe inflammatory states [27]. Thus, inflammation is highly likely to be involved at least in part with APN abnormalities and insulin resistance. Because adipose tissue excessively produces complement C1 in obesity, the complement system may be associated with the inflammation of adipose tissue and insulin resistance [28]. The association with insulin resistance is also suggested by a finding that the C1q-APN/total-APN ratio is a useful marker for metabolic syndrome [12]. Accordingly, the high C1q-APN/total-APN ratios in Japanese–Americans may indicate that APN binds to the inflammatory molecule C1q and inhibits inflammation to play a protective role in the chronic inflammatory state resulting from an American lifestyle.

## Study limitations

In this study, we did not evaluate high-molecular weight (HMW)-APN which is the most biologically active form of APN. A previous report showed that the serum HMW-APN level and the C1q/HMW-APN ratio were independent markers of coronary artery stenosis [29]. Another limitation of this study was that its cross-sectional study design does not clearly show causal relationships. To investigate the direct impact of westernized lifestyles, future prospective longitudinal studies are necessary. In addition to westernized lifestyles, APN characteristics may be influenced by various factors such as genetic [30] and so on. Furthermore, this study included only native Japanese and Japanese–Americans but did not examine other ethnic groups.

## Conclusions

In Asians including Japanese, even mild obesity poses a risk of DM [31]. As one of the causes of the increased incidence of DM in Japanese, in whom the prevalence of obesity is lower than in Europeans and Americans, the results of this study suggest that the westernization of their lifestyles might have caused both quantitative and qualitative changes in APN, which might have resulted in the induction of insulin resistance and increased risk for the development of DM.

## Additional file

**Additional file 1: Table A.** Relationships of Matsuda Index by regression analysis with total APN (A) and C1q-APN/total-APN ratio (B) as the dependent variables in native Japanese and Japanese-Americans.

## Abbreviations

APN: adiponectin
C1q-APN: C1q-adiponectin complex
DM: diabetes mellitus
CRP: C-reactive protein
OGTT: oral glucose tolerance test
BMI: body mass index
IRI: immunoreactive insulin
HDL: high-density lipoprotein
LDL: low-density lipoprotein
ELISA: enzyme-linked immunosorbent assay
NGT: normal glucose tolerance
IGT: impaired glucose tolerance
AUC: area under the curve
HMW-APN: high-molecular weight adiponectin
