# Supplementary material for: Westernization of lifestyle affects quantitative and qualitative changes in adiponectin
Source: Cardiovasc Diabetol. 2017 Jul 6;16:83. doi: 10.1186/s12933-017-0565-z (PMC5501538; doi:10.1186/s12933-017-0565-z)
Supplement: Supplementary file 1 — Additional file 1: Table A. Relationships of Matsuda Index by regression analysis with total APN (A) and C1q-APN/total-APN ratio (B) as the dependent variables in native Japanese and Japanese-Americans. [file 12933_2017_565_MOESM1_ESM.docx]

**Table A‒**Relationships of log Matsuda Index by regression analysis with log total APN (A), and log C1q-APN/total-APN ratio (B) as the dependent variables in native Japanese and Japanese-Americans.

β, Standardized regression coefficients.
